# Maternal perinatal depressive symptoms and offspring psychotic experiences at 18 years of age: a longitudinal study

**DOI:** 10.1016/S2215-0366(20)30132-2

**Published:** 2020-05

**Authors:** Ramya Srinivasan, Rebecca M Pearson, Sonia Johnson, Gemma Lewis, Glyn Lewis

**Affiliations:** a Division of Psychiatry, Faculty of Brain Sciences, University College London, London, UK; b Population Health Sciences, University of Bristol, Bristol, UK

## Abstract

**Background:**

Evidence exists that maternal depression in the perinatal period has an adverse effect on a range of early childhood outcomes and increases the risk of offspring depression during adolescence. However, the association between maternal depression during the perinatal period and offspring psychotic experiences has not been investigated. We aimed to investigate whether there is an association between maternal antenatal or postnatal depression and offspring psychotic experiences at 18 years of age.

**Methods:**

This longitudinal study used data from the Avon Longitudinal Study of Parents and Children (ALSPAC), a prospective birth cohort, which recruited 14 541 pregnant women with an estimated delivery date between April 1, 1991, and Dec 31, 1992. Perinatal depression was measured using the Edinburgh Postnatal Depression Scale (EPDS); offspring psychotic experiences at 18 years of age were measured using the Psychosis-Like Symptom Interview. Offspring of mothers with complete data on maternal perinatal depression measures, and complete data on outcome (psychotic experiences) and confounding variables were included in the main analysis. For the main analysis, we used logistic regression to examine the associations between maternal depression (antenatal and postnatal) and offspring psychotic experiences at the age of 18 years. We used biprobit regression to model the association between maternal antenatal depression and the two offspring outcomes (psychotic experiences and depression) at 18 years of age jointly.

**Findings:**

3067 offspring for whom data were available on maternal perinatal depression and offspring psychotic experiences aged 18 years were included in analyses. Maternal antenatal depressive symptoms were associated with offspring psychotic experiences at 18 years of age, with an unadjusted odds ratio (OR) of 1·38 (95% CI 1·18–1·61, p=0·0001) and after adjustment for confounders, an OR of 1·26 (1·06–1·49, p=0·0074). Maternal antenatal depressive symptoms were associated with both offspring psychotic experiences at the age of 18 years (n=2830, OR for a 5-point increase in EPDS score: 1·32 [95% CI 1·16–1·51], p<0·0001) and offspring depression at 18 years (OR for a 5-point increase in EPDS score: 1·18 [1·03–1·34], p=0·016). From joint modelling, there was no evidence that the association between maternal antenatal depression and offspring psychotic experiences differed in strength compared with offspring depression (p=0·19).

**Interpretation:**

The offspring of mothers who experience depression in the perinatal period are more likely to report psychotic experiences at 18 years of age. If the association is found to be causal, it would strengthen the case for identifying and treating maternal depression during and after pregnancy.

**Funding:**

UK Medical Research Council and the Wellcome Trust.

## Introduction

Increasing evidence suggests that psychosis is best viewed as a continuum and that psychotic experiences not meeting criteria for psychotic disorder are much more common in both the adolescent and adult populations than previously realised.^1^ Psychotic experiences could represent the milder end of the psychosis continuum. Children and adults who report psychotic experiences during childhood are at higher risk of developing a psychotic disorder such as schizophrenia during adulthood.^1^ There is also evidence that psychotic experiences are not just related to psychotic disorders but are also associated with more severe presentations of common mental health disorders such as anxiety and depression.^2^ Thus, the study of psychotic experiences is relevant to both the early phases of development of psychotic disorders^1^ and the more severe presentations of common mental health disorders.

Maternal perinatal depression is known to have an adverse effect on several aspects of child development, including social, emotional, and cognitive function,^3^, ^4^ and is associated with offspring depressive symptoms in adolescence^5^ and adulthood.^6^ The neurodevelopmental hypothesis of schizophrenia proposes that abnormal brain development might contribute to the development of the disorder.^7^ Maternal perinatal depression is a form of psychological distress and might reflect chronic maternal stress, which could affect neurodevelopment of offspring,^3^ increasing offspring risk of psychotic experiences. Antenatal depression might affect fetal neurodevelopment in utero and have epigenetic effects on the fetus.^3^ In addition, antenatal depression might have effects on postnatal bonding and parenting. Maternal postnatal depression might negatively affect bonding and parenting during infancy, which might affect offspring attachment style^4^ and increase the risk of psychotic experiences.^8^ If a causal association were to be found between maternal perinatal depression and offspring psychotic experiences, there is the potential for intervention during this crucial period to reduce not only maternal distress but also offspring risk in the future.

**Research in context**

**Evidence before this study**

Maternal depression during pregnancy and the postnatal period are known to be associated with adverse effects on a range of early childhood outcomes and to increase the risk of offspring depression. We searched PubMed for population-based cohort studies published in English before Nov 1, 2019, using the search terms (“antenatal” OR “prenatal” OR “pregnan*” OR “postnatal” OR “perinatal”) AND “depress*” AND (“mother” OR “maternal” OR “women”) AND (“offspring” OR “child” OR “adolesc*”) AND (“psycho*” OR “schiz*”). We found one study that reported an association between maternal antenatal depression and schizophrenia in adult offspring at approximately 30 years of age, but only in those with a parental history of psychotic disorder. Similar results were found in subsequent analyses of the same cohort, when offspring were approximately 43 years of age. Another study found no association between maternal antenatal depression and depressive and schizotypal traits in adult offspring. These studies were limited by the quality of the assessment of maternal antenatal depression and were all based on the same cohort. No previous study had investigated the association between maternal perinatal depression and offspring psychotic experiences during adolescence.

**Added value of this study**

In this analysis of a prospective birth cohort, we found that maternal depression during pregnancy and the postnatal period is associated with offspring psychotic experiences during adolescence. This association is independent of the association with offspring depression. These findings represent an important addition to the scientific literature; the findings expand the range of offspring outcomes associated with perinatal depression and suggest that common mechanisms might underlie the risk of many psychiatric disorders.

**Implications of all the available evidence**

Maternal perinatal depression affects a range of offspring outcomes beyond early childhood. This emphasises the importance of identification and treatment of maternal depression during this crucial period.

To our knowledge, no previous studies have examined the association between maternal perinatal depression and offspring psychotic experiences. One study has investigated maternal perinatal depression and offspring schizophrenia in a Finnish cohort.^9^ Initial findings reported that maternal depression during pregnancy was associated with schizophrenia in adult offspring,^9^ but subsequent analyses suggested that this association was observed only in those with a family history of psychotic disorder.^10^ However, maternal antenatal depression did seem to have a potentiating effect on the genetic risk for schizophrenia, whereby the risk for schizophrenia was much higher in the offspring of mothers with antenatal depression and a family history of psychosis than in the offspring with only a family history of psychosis.^10^ Similar findings have been found in a subsequent follow-up study.^11^ Another study in the same cohort found no association between maternal antenatal depression and offspring affective and schizotypal traits during adulthood;^12^ surprisingly, this study also found no association between familial risk of psychosis and offspring schizotypal traits. These studies are, however, limited by the fact that maternal antenatal depression was based on a single self-report question and the measures of affective and schizotypal traits were based on self-report questionnaires; there is some evidence that semi-structured interviews are better at finding schizotypal traits than self-report questionnaires.^13^

We aimed to investigate whether there is an association between maternal antenatal or postnatal depression and offspring psychotic experiences at 18 years of age. Maternal perinatal depression is associated with depressive symptoms in offspring during adolescence, and there is evidence that psychotic experiences are often comorbid with depressive symptoms.^14^ Therefore, we also aimed to examine the extent to which there is a differential association between maternal depression and offspring psychotic experiences and depression.

## Methods

### Study design and participants

In this longitudinal study, we used a sample of participants from the Avon Longitudinal Study of Parents and Children (ALSPAC) cohort. All pregnant women living in the former Avon Health Authority of southwest England with an estimated delivery date between April 1, 1991, and Dec 31, 1992, were invited to participate in ALSPAC. The parents and children of 14 541 pregnancies were initially recruited.^15^, ^16^ Detailed data about the cohort have been collected since early pregnancy, including self-reported information from mothers and children, and face-to-face assessment in research clinics.

The starting sample for this study comprised all mothers with singleton pregnancies with complete data on all four maternal antenatal and postnatal depression measures (two antenatal and two postnatal). From this starting sample, adolescents with complete data across all exposure (four maternal depression measures), outcome (psychotic experiences), and confounding variables were included in this study.

Ethical approval for the study was obtained from the ALSPAC Law and Ethics Committee and the Local Research Ethics Committee. Consent for biological samples has been collected in accordance with the Human Tissue Act (2004). Participants gave written informed consent.

### Procedures

The Psychosis-Like Symptom Interview (PLIKSi) is a semi-structured interview based on the principles of the Schedule for Clinical Assessment in Neuropsychiatry.^17^ The PLIKSi covers key core psychotic experiences, including hallucinations, delusions, and experiences of thought interference and passivity phenomena. Questions about each experience started with a structured stem asking whether, from the age of 12 years, the participant had ever had the experience. Participants who responded “yes” or “maybe” were then asked further questions to establish whether the experience was psychotic. Coding of psychotic experiences followed glossary definitions and rating rules for the Schedule for Clinical Assessment in Neuropsychiatry. Interviewers rated experiences as being not present, suspected, or definitely psychotic. Experiences were rated as being definitely psychotic only when a clear example was provided, and unclear responses were always down rated. Interviews were administered by trained psychology graduates who were masked to previous assessments. A psychiatrist rated samples of recorded interviews to ensure that ratings were correct. Inter-rater reliability of the PLIKSi in ALSPAC at the age of 18 years was high (κ=0·83).^17^ Test–retest reliability was assessed using 162 individuals who were reassessed after 47 days (κ=0·76), 46 of whom were assessed by the same interviewer (κ=0·86).^17^

Total scores from the PLIKSi were recoded into a binary variable indicating absence, or suspected or definite presence of psychotic symptoms that were not attributable to sleep or fever. This approach is consistent with previous investigations of psychotic experiences in ALSPAC^14^, ^17^ and is due to the positive skew (with most participants scoring 0, indicating no psychotic experiences). We did a sensitivity analysis using a binary variable indicating the absence or presence of psychotic symptoms that would meet the criteria for a clinical disorder based on previous work in ALSPAC (appendix pp 1, 3).^17^

Depression in offspring at 18 years of age was measured with the computerised version of the Clinical Interview Schedule-Revised (CIS-R),^18^ which derives a diagnosis of depression according to ICD-10 criteria.^18^ The interview is fully standardised and reliable whether done by a trained interviewer or self-administered on the computerised version.^18^, ^19^ The CIS-R is designed for, and has been widely used in, community samples, including the National Surveys of Psychiatric Morbidity.^20^ A binary variable indicating a primary diagnosis of major depression on the CIS-R was used to examine the associations between offspring depression and psychotic experiences at 18 years of age.

Symptoms of maternal perinatal depression were measured with the Edinburgh Postnatal Depression Scale (EPDS). The EPDS is a ten-item self-report depression questionnaire validated for use in the perinatal periods.^21^ It is also validated for use outside the perinatal period and in men.^5^, ^22^ Scores of more than 12 have a high sensitivity and specificity for major depressive disorder.^5^, ^21^ This study primarily used the continuous scores to make full use of the variation in symptoms, although the threshold of more than 12 was used to confirm the results of the main analyses. For the purposes of this study, the antenatal period was considered as being during pregnancy and the postnatal period was considered the first year postnatally.

Postal questionnaires, including the EPDS, were administered to mothers at approximately 18 and 32 weeks antenatally, and at 8 weeks and 8 months postnatally. EPDS was also administered to mothers to assess for depression during their child's life on six occasions between the ages of 1 year and 12 years.

To obtain the antenatal depression measure, the mean EPDS score was calculated by combining those from the two antenatal periods as used previously.^5^, ^23^ The maternal postnatal depression score was the mean of the 8 weeks and 8 months postnatal EPDS scores, as used previously.^5^, ^23^ Results for the association between antenatal and postnatal depression and offspring outcomes are presented for a 5-point increase in EPDS score (ie, approximately 1 SD) throughout.

To account for repeated exposure throughout a child's life, a count of subsequent maternal depressive episodes was derived (ie, the numbers of times a mother scores >12 on any of the six EPDS measures from the age of 1 year to 12 years). This measure is valid only if all timepoints are included, and most women missed one or two EPDS questionnaires; therefore, this measure was not included in the complete case analysis because it restricted the sample size.

We adjusted the main analysis for maternal age at delivery, maternal social class, maternal education, maternal parity, whether the pregnancy was intentional or not, maternal smoking in the first trimester of pregnancy, maternal cannabis use in the first trimester, maternal infection during pregnancy, maternal family history of schizophrenia, maternal family history of depression, and offspring sex. Covariates were selected on the basis of previous reports of their association with maternal perinatal depression and with psychosis.^5^, ^14^, ^24^ Factors that might be on the causal pathway (ie, occurring after birth and during childhood, such as offspring experience of abuse or bullying) were not included. Maternal education was coded 1 to 5 ranging from minimal education to university level education, and was dichotomised to create a binary variable with education up to the age of 16 years categorised as low education and education beyond 16 years categorised as high. Social class was measured using five categories from the 1991 classification of the UK Office of Population Censuses and Surveys, and was dichotomised into manual and non-manual.

Supplementary analyses adjusting for family history and adjusting for offspring schizophrenia polygenic risk score were also done (appendix pp 1–2, 5–6).

Fathers also completed the EPDS at 18 weeks of pregnancy (paternal antenatal depression) and 8 months postnatally (paternal postnatal depression). Paternal education was coded 1 to 5, ranging from minimal education to university level. Therefore, the association between paternal antenatal and postnatal depression scores with offspring psychotic experiences was also investigated.

### Statistical analysis

In the main analysis, we investigated the association between antenatal depression and psychotic experiences in offspring by 18 years of age and between postnatal depression and psychotic experiences using logistic regression. We then repeated analyses, adjusting for confounding variables. As sensitivity analyses, the main analyses were repeated with the antenatal and postnatal EPDS scores recoded as a binary exposure, and looking at the effects of each of the two antenatal EPDS measures and each of the two postnatal EPDS measures separately. Secondary analyses included analyses of effect modification by later maternal depression, and the investigation of relationship between paternal perinatal depression and offspring psychotic experiences. Several post-hoc sensitivity analyses examined the effect of family history of mental disorder as a confounder of the association between maternal perinatal depression and offspring psychotic experiences. See the appendix (pp 1–2, 5–6) for details.

To explore the association between maternal antenatal and postnatal depression, we first assessed the correlation between them, and did a principal components analysis, the results of which are presented in the appendix (pp 2, 4). We also examined the effects of persistent depression during the perinatal period; the details of this analysis are presented in the appendix (pp 1, 4).

To investigate whether later episodes of maternal depression occurring during the child's life are an effect modifier of the association between maternal antenatal depression and offspring psychotic experiences by 18 years of age, models testing the association between antenatal depression scores and offspring psychotic experiences were adjusted for the number of later episodes of maternal depression and then tested for an interaction between maternal antenatal depression and later maternal depression. We did a sensitivity analysis to test the association between maternal antenatal depressive symptoms and offspring psychotic experiences adjusting for the most recent measurement of maternal depression (when offspring were 12 years of age) and then tested for an interaction between maternal antenatal depressive symptoms and maternal depression when offspring were aged 12 years. These analyses were repeated with maternal postnatal depressive symptoms as the exposure and are reported in the appendix (pp 4–5).

We used logistic regression to investigate the association between paternal antenatal and postnatal depression scores and offspring psychotic experiences at 18 years of age. Because of the limited availability of data on paternal depression in the perinatal period, this analysis was only adjusted for paternal education based on previous literature on the effects of paternal perinatal depression and offspring depression at the age of 18 years.^5^

To investigate the association between offspring psychotic experiences by the age of 18 years, offspring depression at the age of 18 years, and maternal antenatal depression, we first assessed the correlation between offspring psychotic experiences and offspring depression, and then used biprobit regression. The biprobit method allows joint modelling of psychotic experiences and depression at 18 years of age and testing of equality of regression parameters that express the effect of the respective exposure (ie, antenatal depression) on each outcome (ie, psychotic experiences and depression) using a likelihood ratio test. To enable easier interpretation, we converted probit estimates into odds ratios (ORs) by obtaining approximations of the logit parameters by multiplying the probit parameters by a factor of 1·6.^14^ p values of less than 0·05 were considered to be significant.

We did our main analyses using a sample with complete data on exposure, outcome, and confounding variables. The characteristics of mothers and offspring in the sample with complete data and of those with missing data were compared using Student's *t* test and the χ^2^ test. Missing data were assumed to be associated with observed data (ie, missing at random).^25^ When data are missing at random, analyses based on complete cases might be biased.^25^ We therefore did sensitivity analyses using multiple imputation with chained equations in two ways. First, we replaced missing data in the outcome and confounder variables for all participants who had complete data on the outcome at 12 years of age. Second, we replaced missing data in the outcome and confounder variables for all participants with complete exposure data. We imputed 100 datasets. To predict missing data, all variables included in the analysis models, and a number of auxiliary variables, were included across 100 imputed datasets. Analyses were then run across imputed datasets using the mi estimate command, which fits a model to each of the imputed datasets and pools individual results to obtain a pooled estimate derived using Rubin's combination rules. All analyses were done in StataSE (version 15).

### Role of the funding source

The funders of the study had no role in the study design; in the collection, analysis, and interpretation of data; in the writing of the report; or in the decision to submit the Article for publication. All authors declare independence from the funding sources. The corresponding author had full access to all the data in the study and had final responsibility for the decision to submit for publication.

## Results

Complete exposure measures (two antenatal and two postnatal EPDS score) were available for 9204 mothers (figure; table 1). Data on maternal depression during pregnancy and the postnatal period and data on confounding variables were available for 3067 adolescents who had completed the PLIKSi. Participants with missing data were more likely to be mothers with higher antenatal EPDS scores, higher postnatal EPDS scores, of manual social class, with less education, who had smoked during the first trimester of pregnancy, who had an infection during pregnancy, and who had male offspring.

**Figure:**
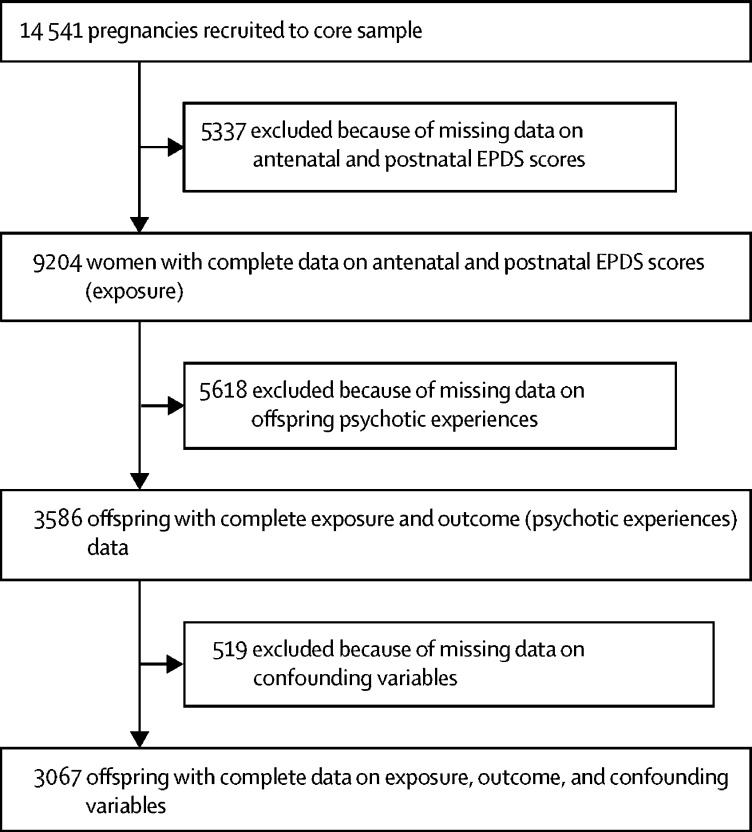
Study profile EPDS=Edinburgh Postnatal Depression Scale.

**Table 1:** General characteristics of the study population

		**Mothers with complete exposure measures (n=9204)**	**Offspring with all exposure, outcome, and confounding variables (n=3067)**	**Offspring with missing data (n=6137)**	p value*
Maternal antenatal EPDS scores		6·7 (4·4; 0–29)	6·1 (4·1; 0–23)	7·0 (4·5; 0–29)	<0·0001
	Score >12	1079 (12%)	257 (8%)	822 (13%)	<0·0001
Maternal postnatal EPDS scores		5·6 (4·2; 0–27)	5·2 (3·9; 0–25)	5·8 (4·3; 0–27)	<0·0001
	Score >12	691 (8%)	170 (6%)	521 (8%)	<0·0001
Maternal age at delivery, years		28·7 (4·64; 15–44)	29·6 (4·39; 16–44)	28·3 (4·70; 15–44)	<0·0001
Maternal parity		0·81 (0·93; 0–8)	0·66 (0·81; 0–5)	0·89 (0·98; 0–8)	<0·0001
Maternal ethnicity		..	..	..	0·21
	White	8963/9132 (98%)	3015/3064 (98%)	5948 (98%)	..
	Ethnic minority	169/9132 (2%)	49/3064 (2%)	120 (2%)	..
Maternal social class		..	..	..	<0·0001
	Non-manual	6484/7923 (82%)	2629 (86%)	3855/4856 (79%)	..
	Manual	1439/7923 (18%)	438 (14%)	1001/4856 (21%)	..
Maternal education		..	..	..	<0·0001
	Non-compulsory	3475/9164 (38%)	1560 (51%)	1915/3067 (31%)	..
	Compulsory only	5689/9164 (62%)	1507 (49%)	4182/3067 (69%)	..
Pregnancy intentional or not		..	..	..	<0·0001
	No	2445/9169 (27%)	664 (22%)	1781/6102 (29%)	..
	Yes	6724/9169 (73%)	2403 (78%)	4321/6102 (71%)	..
Maternal smoking in the first trimester		..	..	..	<0·0001
	No	7263 (79%)	2631 (86%)	4632 (75%)	..
	Yes	1941 (21%)	436 (14%)	1505 (25%)	..
Maternal cannabis use in the first trimester		..	..	..	0·091
	No	8645/8819 (98%)	3017 (98%)	5628/5752 (98%)	..
	Yes	174/8819 (2%)	50 (2%)	124/5752 (2%)	..
Maternal infection during pregnancy		..	..	..	0·40
	No	6886/9201 (75%)	2321 (75%)	4547/6134 (75%)	..
	Yes	2315/9201 (25%)	755 (25%)	1560/6134 (25%)	..
Maternal family history of schizophrenia		..	..	..	0·36
	No	9014/9071 (99%)	3051 (99%)	5963/6004 (99%)	..
	Yes	57/9014 (1%)	16 (1%)	41/6004 (1%)	..
Maternal family history of depression		..	..	..	0·010
	No	6778/9071 (75%)	2342 (76%)	4436/6004 (74%)	..
	Yes	2293/9071 (25%)	725 (24%)	1568/6004 (26%)	..
Maternal history of depression		..	..	..	<0·0001
	No	8331/9071 (92%)	2891 (94%)	5440/6004 (91%)	..
	Yes	740/9071 (8%)	176 (6%)	564/6004 (9%)	..
Maternal history of schizophrenia		..	..	..	0·68
	No	9073/9083 (100%)	3063 (100%)	6010/6016 (100%)	..
	Yes	10/9083 (<1%)	4 (<1%)	6/6016 (<1%)	..
Offspring sex		..	..	..	<0·0001
	Male	4736 (51·5)	1361 (44%)	3375 (55%)	..
	Female	4468 (48·5)	1706 (56%)	2762 (45%)	..
Offspring depression aged 18 years		..	..	..	0·048
	No	3196/3460 (92%)	2626/2830 (93%)	570/630 (90·5)	..
	Yes	264/3460 (8%)	204/2830 (7%)	60/630 (9·5)	..
Offspring psychotic experiences aged 18 years		..	..	..	0·89
	None	3322/3586 (93%)	2842 (93%)	480/519 (92%)	..
	Suspected or definite	264/3586 (7%)	225 (7%)	39/519 (8%)	..

Data are mean (SD; range), n (%), or n/N (%). EPDS=Edinburgh Postnatal Depression Scale.

* Comparison between participants with complete data and those with missing data.

The mean maternal depression score was 6·7 (SD 4·4; range 0–29) during the antenatal period and 5·6 (4·2; 0–27) during the postnatal period (table 1). Measures of maternal depression were highly correlated with each other. The correlation between depressive symptoms across the two antenatal measurements at 18 and 32 weeks of pregnancy was 0·64, across the two postnatal measurements at 8 weeks and 8 months postnatally was 0·61, and between the antenatal mean and postnatal mean was 0·66. 1079 (12%) of 9204 mothers exceeded thresholds for depression antenatally (means scores of >12 across the two antenatal timepoints), and 691 (8%) mothers exceeded these thresholds postnatally.

At 18 years of age, 4248 adolescents completed the CIS-R. Of these adolescents, 334 (8%) met criteria for ICD-10 depression. Data on PLIKSi and depression at the age of 18 years were available for 4051 adolescents, with 71 (2%) meeting criteria for depression and being identified as having experienced suspected or definite psychotic experiences. Data on maternal antenatal and postnatal depression scores were available for 3302 adolescents with data on CIS-R and PLIKSi at the age of 18 years, of whom 51 (2%) met criteria for depression and suspected or definite psychotic experiences. Offspring depression at the age of 18 years was positively but weakly correlated with offspring psychotic experiences at the age of 18 years (r=0·16, p<0·0001).

We found evidence that the offspring of mothers with higher antenatal EPDS score were more likely to have had psychotic experiences at the age of 18 years (OR for a 5-point increase in EPDS score: 1·38 [95% CI 1·18–1·61], p=0·0001; table 2). The evidence for this association remained after adjustment for covariates (OR for a 5-point increase in EPDS score: 1·26 [95% CI 1·06–1·49], p=0·0074). These results were similar, although the strength of the association was attenuated, when using binary antenatal depression variable derived using the clinical threshold of EPDS scores of more than 12, both in the univariable model (OR 1·85 [95% CI 1·24–2·77], p=0·0028) and after adjustment for covariates (1·49 [0·98–2·28], p=0·065). Antenatal EPDS scores for each timepoint had similar effects to those reported for averaged antenatal depression scores (appendix p 3).

**Table 2:** Association between maternal antenatal depression and offspring psychotic experiences by the age of 18 years in a sample with complete exposure, outcome, and confounding variables

	**Mothers with complete exposure data (n=9204)**	**Offspring with complete exposure, outcome, and confounding variable data (n=3067)**	**Offspring with psychotic experiences aged 18 years (n=3067)**	**Model 1**		**Model 2**	
				Odds ratio (95% CI)	p value	Odds ratio (95% CI)	p value
Low maternal antenatal depressive symptoms score (0–5·5)	4336 (47%)	1596 (52%)	92 (3%)	1 (ref)	..	1 (ref)	..
Moderate maternal antenatal depressive symptoms score (6–12)	3789 (41%)	1214 (40%)	102 (3%)	1·49 (1·12–2·00)	0·0066	1·37 (1·01–1·84)	0·041
High maternal antenatal depressive symptoms score (>12)	1079 (12%)	257 (8%)	31 (1%)	2·24 (1·46–3·45)	0·0002	1·77 (1·12–2·79)	0·015
Antenatal maternal depression score (5-point increase in EPDS score)	..	..	..	1·38 (1·18–1·61)	0·0001	1·26 (1·06–1·49)	0·0074

Model 1 shows univariable associations between each timing of maternal perinatal depression and offspring psychotic experiences at the age of 18 years. Model 2 is model 1 including confounding variables: maternal age at delivery, maternal social class, maternal education, maternal parity, whether the pregnancy was intentional or not, maternal smoking in the first trimester, maternal cannabis use in the first trimester, maternal infection during pregnancy, maternal family history of schizophrenia, maternal family history of depression, maternal history of depression before pregnancy, and offspring sex. EPDS=Edinburgh Postnatal Depression Scale.

There was evidence of an association between maternal postnatal depression score and offspring psychotic experiences at the age of 18 years (OR for a 5-point increase in EPDS score: 1·25 [95% CI 1·06–1·47], p=0·0067). The evidence for this association remained but was attenuated after adjusting for covariates (OR for a 5-point increase in EPDS score: 1·17 [0·99–1·39], p=0·065). These results were replicated using the binary postnatal depression variable derived using the clinical threshold of EPDS scores or more than 12, in the univariable model (OR 2·09 [95% 1·31–3·31], p=0·0018) and evidence of an association remained after adjusting for covariates (1·81 [95% CI 1·12–2·93], p=0·016). The results of analyses using the postnatal EPDS scores for each timepoint were similar for maternal postnatal depression measured at 8 weeks, but no evidence of an association was found for maternal depression at 8 months postnatally (appendix p 3). Sensitivity analyses adjusting for a range of family history variables (including family history of bipolar disorder, family history of mental health admission, paternal family history of psychosis, and schizophrenia polygenic risk score) as confounders showed no effect (appendix pp 4–5).

When considering the effect of maternal depression later in the child's life, there was evidence of an association between maternal antenatal depressive symptoms and offspring psychotic experiences by the age of 18 years in the sample with complete data on maternal perinatal depressive symptoms and maternal depression when offspring were 12 years of age (n=2663, OR for a 5-point increase in EPDS score: 1·32 [95% CI 1·11–1·58], p=0·0016). The association between antenatal depression score and offspring psychotic experiences was independent of the most recent measurement of maternal depression when offspring were 12 years of age (OR for a 5-point increase in EPDS score: 1·27 [1·02–1·52], p=0·013). There was no evidence that the association between maternal antenatal depressive symptoms differed according to maternal depression when offspring were 12 years of age (p_interaction_=0·83).

There was evidence of an association between maternal antenatal depressive symptoms and offspring psychotic symptoms by the age of 18 years in the sample with complete data on maternal perinatal depressive symptoms and later maternal depressive episodes (n=2126, OR for a 5-point increase in EPDS score: 1·32 [95% CI 1·09–1·61], p=0·0052). The association between antenatal depression and offspring psychotic experiences was independent of the number of later maternal depressive episodes (OR for a 5-point increase in EPDS score: 1·32 [95% CI 1·02–1·71], p=0·033). There was no evidence that the association between maternal antenatal depressive symptoms differed according to the number of later maternal depressive episodes (p_interaction_=0·61).

With regard to paternal antenatal and postnatal depression, in univariable and adjusted models, a 5-point increase in paternal antenatal EPDS score (unadjusted: n=2404, OR 1·17 [95% CI 0·95–1·44], p=0·15; adjusted: n=2404, 1·08 [0·87–1·35], p=0·47) or paternal postnatal EPDS score (unadjusted: n= 404, 1·08 [0·86–1·35], p=0·52; adjusted: n=2404, 1·06 [0·84–1·34], p=0·61) was not associated with offspring psychotic experiences at the age of 18 years. These analyses were also done using paternal EPDS scores as a binary variable derived using the clinical threshold of more than 12; the results from these analyses are presented in the appendix (p 5).

Results of the biprobit analyses found evidence for an association between maternal antenatal depressive symptoms and both offspring psychotic experiences at the age of 18 years (n=2830, OR for a 5-point increase in EPDS score: 1·32 [95% CI 1·16–1·51], p<0·0001) and offspring depression at 18 years (OR for a 5-point increase in EPDS score: 1·18 [1·03–1·34], p=0·016). When the sizes of the associations were compared, there was no evidence that the strength of association differed for psychotic experiences or depression (p=0·19).

Results for the association between maternal antenatal depression scores and offspring psychotic experiences at 18 years of age based on multiple imputation are shown in Table 3, Table 4. A similar pattern of associations between maternal antenatal and postnatal depression scores and offspring psychotic experiences at the age of 18 years was observed in both of the imputation analyses in comparison to the complete case sample. There remained evidence for an association between antenatal depression and offspring psychotic experiences at the age of 18 years in the univariable model and after adjustment for covariates.

**Table 3:** Association between maternal antenatal depression and offspring psychotic experiences by the age of 18 years in a sample with imputed data

	**Mothers with complete exposure data (n=9204)**	**Mothers with complete exposure data in imputed sample (n=5577)**	**Model 1**		**Model 2**	
			Odds ratio (95% CI)	p value	Odds ratio (95% CI)	p value
Low maternal antenatal depressive symptoms score (0–5·5)	4336 (47%)	2779 (50%)	1 (ref)	..	1 (ref)	..
Moderate maternal antenatal depressive symptoms score (6–12)	3789 (41%)	2227 (40%)	1·45 (1·12–1·89)	0·0052	1·31 (1·00–1·71)	0·052
High maternal antenatal depressive symptoms score (>12)	1079 (12%)	571 (10%)	2·2 (1·55–3·26)	<0·0001	1·69 (1·13–2·52)	0·011
Antenatal maternal depression (5-point increase in EPDS score)	..	..	1·37 (1·19–1·58)	<0·0001	1·22 (1·05–1·43)	0·0094

Model 1 is univariable associations between each timing of maternal perinatal depression and offspring psychotic experiences at the age of 18 years. Model 2 is model 1 adjusted for confounding variables: maternal age at delivery, maternal social class, maternal education, maternal parity, whether the pregnancy was intentional or not, maternal smoking in the first trimester, maternal cannabis use in the first trimester, maternal infection during pregnancy, maternal family history of schizophrenia, maternal family history of depression, maternal history of depression before pregnancy, and offspring sex. EPDS=Edinburgh Postnatal Depression Scale.

**Table 4:** Association between maternal antenatal depression and offspring psychotic experiences by the age of 18 years in a sample with imputed data

	**Mothers with complete exposure data (n=9204)**	**Model 1**		**Model 2**	
		Odds ratio (95% CI)	p value	Odds ratio (95% CI)	p value
Low maternal antenatal depressive symptoms score (0–5·5)	4336 (47%)	1 (ref)	..	1 (ref)	..
Moderate maternal antenatal depressive symptoms score (6–12)	3789 (41%)	1·42 (1·14–1·79)	0·0023	1·24 (0·98–1·57)	0·076
High maternal antenatal depressive symptoms score (>12)	1079 (12%)	2·19 (1·55–3·09)	<0·0001	1·60 (1·10–2·33)	0·014
Antenatal maternal depression (5-point increase in EPDS score)	..	1·36 (1·19–1·56)	<0·0001	1·20 (1·03–1·40)	0·019

Model 1 shows univariable associations between each timing of maternal perinatal depression and offspring psychotic experiences at the age of 18 years. Model 2 is model 1 adjusted for confounding variables: maternal age at delivery, maternal social class, maternal education, maternal parity, whether the pregnancy was intentional or not, maternal smoking in the first trimester, maternal cannabis use in the first trimester, maternal infection during pregnancy, maternal family history of schizophrenia, maternal family history of depression, maternal history of depression before pregnancy, and offspring sex. EPDS=Edinburgh Postnatal Depression Scale.

## Discussion

To our knowledge, this study is the first to examine the association between maternal depressive symptoms during the perinatal period and offspring psychotic experiences at 18 years of age. We found evidence that the offspring of mothers with higher perinatal depressive symptom scores were more likely to report psychotic experiences than the offspring of mothers with lower depressive scores. This finding is consistent with the hypothesis that maternal depression during the perinatal period might be a risk factor for offspring psychotic experiences. The association between maternal antenatal depression score and offspring psychotic experiences was similar in size to that between maternal antenatal depression score and offspring depression at 18 years of age.

The strengths of this study include the longitudinal design, large sample size, long-term follow-up period, and the availability of data on a broad range of confounders. Maternal depression was assessed several times during pregnancy and the postnatal period, and a reliable semi-structured interview was used to assess offspring psychotic experiences. Attrition is a limitation of this study, as with all cohort studies, and can introduce selection bias; however, multiple imputation was used to investigate the likely impact of attrition and did not alter our conclusions. Previous work in ALSPAC has shown that within-cohort associations tend to be valid even when there are differences between the study sample and the target population,^26^ and, when investigating risk factors, a representative sample is not so important as long as the effects of attrition are minimised.^27^ Although this study was of only one national population, we hope that it will generate future research including cross-cultural comparisons. Genetic confounding must be considered because there is evidence of shared genetic liability between psychotic experiences and a range of other psychiatric disorders.^28^ However, adjusting for maternal family history of psychosis in the main analyses and for paternal family history of psychosis in sensitivity analyses showed no effect. Sensitivity analyses for family history of bipolar disorder and history of mental health admission also showed no effect. Maternal depression measurement was by self-report questionnaire; however, EPDS has been validated against longer assessments and any random measurement error would tend to reduce the size of association rather than lead to spurious associations. In keeping with the vast majority of studies using the PLIKSi, this study used the measure as a binary variable. Although it is more statistically appropriate to model data from the PLIKSi as a binary outcome, psychotic experiences are in reality continuous in the general population.

This study was not able to examine the differential effects of maternal antenatal and postnatal depression due to the correlation between them. Antenatal depression might exert an effect through biological mechanisms acting in utero or after birth through effects on parenting. During pregnancy, maternal depression, which might be a reflection of chronic maternal stress, could affect glucocorticoids, which might have effects on placental function, fetal development, epigenetics, and immune function, all of which have been implicated in the aetiology of psychotic disorders.^4^, ^29^ To investigate such potential mechanisms future research could use measures of hypothalamopituitary axis activation, such as cortisol concentration antenatally. Recent findings in the FinnBrain cohort show changes in maternal cortisol concentration^30^ and cytokine profiles^31^ associated with antenatal depressive symptom trajectories. A mechanism involving immune function would be consistent with findings that maternal infection during pregnancy is a potential risk factor for schizophrenia, particularly in the offspring of parents with a psychiatric disorder.^32^ Maternal depression during pregnancy has also been linked to an increased risk of pregnancy and delivery complications,^4^ which have been linked to psychotic experiences at the age of 12 years in this cohort.^24^ Maternal antenatal depression might also interfere with maternal postnatal bonding with the infant^33^ as well as with maternal representations of the unborn child that are associated with mother–infant interactions^34^ in the early postnatal period.

By contrast, postnatal depression could affect environmental factors such as parenting and wider social engagement.^4^ Infancy is a period of extreme dependency on the caregiver as well as rapid development and is a vulnerable period during development. Psychotic experiences are an important area of interest because they not only represent the milder end of the psychosis continuum and thereby psychotic disorders^1^ but are also associated with more severe presentations of depression and anxiety.^2^

Our evidence suggests that mothers with depression during the perinatal period are more likely to have offspring with psychotic experiences during their adolescence. Our study extends the growing list of childhood and adolescent outcomes associated with perinatal depression. It suggests that common developmental mechanisms might underlie the risk of many psychiatric disorders and adds weight to the importance of identifying and treating maternal mental health problems during pregnancy and the postnatal period, especially in view of evidence that they might be increasing in the current generation of young women.^22^

## Data sharing

Avon Longitudinal Study of Parents and Children data are available to researchers on a confidential and anonymised basis (http://www.bristol.ac.uk/alspac/researchers/access/). STATA code for the analyses is freely available from the corresponding author.
