# Supplementary material for: Maternal perinatal depressive symptoms and offspring psychotic experiences at 18 years of age: a longitudinal study
Source: Lancet Psychiatry. 2020 May;7(5):431–40. doi: 10.1016/S2215-0366(20)30132-2 (PMC7606907; doi:10.1016/S2215-0366(20)30132-2)
Supplement: Supplementary appendix [file mmc1.pdf]

THE LANCET
Psychiatry

Supplementary appendix

This appendix formed part of the original submission and has been peer reviewed. We post it as supplied by the authors.

Supplement to: Srinivasan R, Pearson RM, Johnson S, Lewis G, Lewis G. Maternal perinatal depressive symptoms and offspring psychotic experiences at 18 years of age: a longitudinal study. *Lancet Psychiatry* 2020; **7:** 431–40.

Supplementary Methods

Measures

Adolescent Psychotic Experiences

In addition to the main analyses, analyses were conducted using data from the PLIKSi to classify individuals with regard to whether they would meet criteria for a psychotic disorder based on previous research (1). Individuals were classified as having a psychotic disorder if they reported a definite psychotic experience that was not attributable to sleep or fever that caused severe distress, had a negative impact on social or occupational function or led to help-seeking, and which had occurred at least once per month over the prior 6 months (1). In analyses utilising the PLIKSi to identify those with a psychotic disorder, maternal family history of psychosis could not be included as a confounder due to insufficient data.

Persistent maternal perinatal depression

In order to explore the effects of more persistent maternal perinatal depression on offspring psychotic experiences during adolescence a new categorical variable was created (no depression, depression only antenatally or postnatally, and depression both antenatally and postnatally) using the antenatal and postnatal EPDS scores derived using a clinical cut of EPDS >12. We conducted analyses using this variable as the exposure and then adjusted for the confounders included in the main analyses.

Family History of Mental Health Disorders

In order to better adjust for family history of mental health disorders we conducted the following further analyses.

We conducted analyses, adjusting for paternal family history of schizophrenia, paternal family history of depression and paternal history of depression, in addition to the confounders included in the main analyses. This data was collected in pregnancy via questionnaires that were completed by the mother's partner.

Analyses were also conducted using data on maternal history of mania, paternal history of mania, maternal family history of mania, paternal family history of mania, maternal history of mental health admission, paternal history of mental health admission, maternal family history of mental health admission and paternal family history of mental health admissions. These were included in models in addition to the confounders included in the main analyses except for maternal use of cannabis in the first trimester which could not be included as it was dropped from models which included the additional confounders. This data was collected after via questionnaires that were given to mothers when offspring were aged 18 years and so has been collected after the exposure to maternal perinatal depression.

Schizophrenia Polygenic Risk Score

Analyses adjusting for offspring schizophrenia polygenic risk score (PRS) in addition to the confounders included in the main analyses were conducted. A PRS for schizophrenia was calculated for each ALSPAC individual as previously reported (2) using PLINK (3). The PRS was constructed using genome wide association study (GWAS) summary statistics from discovery studies by summing the number of risk alleles for each single nucleotide polymorphism (SNP) weighted by its effect size in the discovery sample (2). A standardised score generated from a list of SNPs with a genome wide association study (GWAS) discovery sample p-value threshold <0.05.

Statistical analyses

Principal Components Analysis

Principal Components Analysis (PCA) can be used to transform correlated variables into orthogonal (i.e. uncorrelated) factors termed 'principal components'. Two sets of analyses using PCA were conducted.

In the first set of analyses, PCA was used to transform the two exposures (i.e. the antenatal EPDS score and the postnatal EPDS score) into principal components. The principal components from this analysis were used as the exposure in logistic regression models both separately and together. These models were then adjusted for confounders. When conducting this with two correlated measures, the first principal component can be interpreted as representing the average of the antenatal and postnatal scores. The second component is a function of the difference between the two scores. If there is an association between the second component and psychotic experiences, this would support the hypothesis that antenatal and postnatal depressive symptoms have differential effects on the risk of offspring psychotic experiences by the age of 18 years.

In the second set of analyses, PCA was used to transform the EPDS scores from each of the 4 time-points (i.e. 18 weeks antenatally, 32 weeks antenatally, 8 weeks postnatally and 8 months postnatally) into principal components. The primary principal components derived from this analysis were then used as the exposure in the logistic regression model separately, together with the other principal components and was then adjusted for confounders. If there were associations between the additional components and psychotic experiences, this would support the hypothesis that maternal depressive symptoms measured at the different time-points have differential effects on the risk of offspring psychotic experiences.

<u>Supplementary Results</u>

Adolescent psychotic experiences

In the sample with complete exposure and outcome data (n=3586), there were 51 individuals classified as meeting criteria for psychotic disorder based on the PLIKSi. In the sample with data on all exposures, outcomes and confounders (n=3067), 42 individuals met criteria for a psychotic disorder based on the PLIKSi.

There was evidence that mothers with higher antenatal EPDS scores were more likely to have offspring meeting criteria for a psychotic disorder at the age of 18 years (n=3067, unadjusted OR for a 5-point increase in EPDS score: 1.73, 95% CI 1.24 – 2.40, p=0.012). There was weak evidence for this association after adjustment for confounders (adjusted OR for a 5-point increased in EPDS score: 1.42, 95% CI 1.00 – 2.03, p=0.052). There was weak evidence that the offspring of mothers with higher postnatal EPDS scores were more likely to meet criteria for a psychotic disorder by the age of 18 years (n=3067, unadjusted OR for a 5-point increase in EPDS score: 1.35, 95% CI 0.96 – 1.91, p=0.087). The strength of this association was attenuated after adjustment for confounders (adjusted OR for a 5-point increased in EPDS score: 1.18, 95% CI 0.82 – 1.69, p=0.38).

Antenatal depressive symptoms at each time-point and offspring psychotic experiences by the age of 18 years

The EPDS was administered to pregnant women at two antenatal time-points, at 18 and 32 weeks of pregnancy. We found evidence that the offspring of mothers with higher antenatal EPDS scores at 18 weeks' pregnancy were more likely to have experienced psychotic symptoms by the aged of 18 years (unadjusted OR for a 5-point increase in EPDS score: 1.30, 95% CI 1.12 – 1.50, p=0.0005). The evidence for this association remained after adjustment for covariates (OR for a 5-point increase in EPDS score: 1.18, 95% CI 1.01 – 1.38, p=0.032). There was also evidence that the offspring of mothers with higher antenatal EPDS scores at 32 weeks' pregnancy were more likely to have experienced psychotic symptoms by the aged of 18 years (unadjusted OR for a 5-point increase in EPDS score: 1.30, 95% CI 1.13 – 1.49, p=0.0002) . The evidence for this association remained after adjustment for covariates (OR for a 5-point increase in EPDS score: 1.21, 95% CI 1.04 – 1.40, p=0.0085).

Postnatal depressive symptoms at each time-point and offspring psychotic experiences by the age of 18 years

The EPDS was administered to pregnant at two postnatal time-points, at 8 weeks and 8 months postnatally. We found evidence that the offspring of mothers with higher antenatal EPDS scores at 8 weeks after giving birth were more likely to have experienced psychotic symptoms by the aged of 18 years (unadjusted OR for a 5-point increase in EPDS score: 1.27, 95% CI 1.10 – 1.46, p=0.0012) . The evidence for this association remained after adjustment for covariates (OR for a 5-point increase in EPDS score: 1.20, 95% CI 1.04 – 1.39, p=0.016). There was no evidence that higher EPDS scores at 8 months postnatally were associated with offspring psychotic experiences by the age of 18 years (unadjusted OR for a 5-point increase in EPDS score: 1.13, 95% CI 0.98 – 1.31, p=0.10. Adjusted OR for a 5-point increase in EPDS score: 1.07, 95% CI 0.92 – 1.25, p=0.36).

Principal components analysis

PCA of the antenatal EPDS score and postnatal EPDS score showed that the first principal component (PC1) accounted for 84% of the total variance between measurements and that the second component (PC2) accounted for 16% (PC1: eigenvalue 1.68, proportion 0.84. PC2: eigenvalue 0.32, proportion 0.16). There was evidence that PC1 was associated with offspring psychotic experiences by the age of 18 years (unadjusted OR: 1.20, 95% CI 1.09 – 1.32, p=0.0002), and there was evidence for this association after adjusting for confounders (adjusted OR: 1.14, 95% CI 1.03 – 1.27, p=0.013). There was little evidence to suggest that PC2 was associated with offspring psychotic experiences aged 18 years (unadjusted OR: 1.22, 95% CI 0.96 – 1.55, p=0.10; adjusted OR: 1.13, 95% CI 0.89 – 1.43, p=0.33). When both principal components were included in models together there was evidence that PC1 was associated with offspring psychotic experiences by the age of 18 years, but no evidence for PC2 (PC1 unadjusted OR: 1.20, 95% CI 1.09 – 1.33, p=0.0002; PC2 unadjusted OR: 1.21, 95% CI 0.96 – 1.51, p=0.11). There remained evidence for the association between PC1 and offspring psychotic experiences by the age of 18 years after adjustment for confounders (PC1 adjusted OR: 1.14, 95% CI 1.03 – 1.27, p=0.012; PC2 adjusted OR: 1.13, 95% CI 0.90 – 1.42, p=0.30).

PCA of the individual EPDS time-points showed that the first principal component (PC3) accounted for 68% of the total variance between measurements (PC3: eigenvalue 2.73, proportion 0.68. The remaining 3 principal components, PC4, PC5 and PC6, accounted for 13%,, 10% and 9% of the total variance respectively and all had eigenvalues less than 1. There was evidence that PC3 was associated with offspring psychotic experiences by the age of 18 years (unadjusted OR: 1.15, 95% CI 1.07 – 1.25, p=0.0003), and there was evidence for this association after adjusting for confounders (adjusted OR: 1.11, 95% CI 1.02 – 1.20, p=0.014). There was evidence that PC1 was associated with offspring psychotic experiences by the age 18 years after inclusion of the other principal components in the model (PC3 OR: 1.15, 95% CI 1.07 – 1.25, p=0.0003) and after adjustment for confounders (PC3 adjusted OR: 1.11, 95% CI 1.02 – 1.20, p=0.017). There was no evidence for an association between the other principal components (PC4, PC5 and PC6) and offspring psychotic experiences by the age of 18 years in unadjusted and adjusted analyses.

Persistent maternal perinatal depression

There were 255 mothers (8.3%) who scored above 12 on the EPDS at either the antenatally or postnatally; and 86 mothers (2.8%) who scored above 12 on both the antenatal and postnatal mean scores in the sample with complete exposure, outcome and confounding data. The offspring of mothers who were more persistently depressed were more likely to have experienced psychotic symptoms by the age of 18 years (unadjusted OR: 2.67, 95% CI 1.48 – 4.83, p=0.0011). There remained evidence for this association after adjusting for confounders (adjusted OR: 2.13, 95% CI 1.15 – 3.95, p=0.016). In the unadjusted analyses, there was evidence that the offspring of mothers who scored above 12 on either the antenatal or postnatal time-point were more likely to have experienced psychotic experiences by the age of 18 years (unadjusted OR: 1.56, 95% CI 1.01 – 2.40, p=0.044), however there was no evidence for this after adjusting for confounders (adjusted OR: 1.33, 95% CI 0.85 – 2.09, p=0.21).

Maternal postnatal depressive symptoms and later maternal depression

There was evidence of an association between maternal postnatal depressive symptoms and offspring psychotic experiences in the sample with complete data on maternal perinatal depressive symptoms and maternal depression when offspring were 12 years (n=2663, OR for a 5-point increase in EPDS score: 1.21, 95% CI 1.01 – 1.45, p=0.035). There was no evidence of an association between maternal postnatal depressive symptoms and offspring psychotic experiences after including the most recent measurement of maternal depression when offspring were 12 years in the model (OR for a 5-point increase in EPDS score: 1.14, 95% CI 0.94 – 1.38, p=0.18).There was no evidence that this differed according to maternal depression when offspring were 12 years (p-value for the interaction term 0.57).

There was no evidence of an association between maternal perinatal depression and offspring psychotic experiences by the age of 18 years, in the sample with complete data on maternal postnatal depressive symptoms and maternal depressive episodes (n=2126, OR for a 5-point increase in EPDS score: 1.11, 95% CI 0.90 – 1.37, p=0.33). This remained the case after inclusion of the number of later maternal depressive episodes (OR for a 5-point increase in EPDS score: 0.96, 95% CI 0.72 – 1.28, p=0.79).). There was no evidence that this differed according to the number of later maternal depressive episodes (p-value for the interaction term 0.45).

Paternal antenatal and postnatal depression as a binary variable

When examined as a binary variable derived using the clinical threshold of EPDS scores >12, there was little evidence of an association between paternal antenatal EPDS scores and offspring psychotic experiences by the age of 18 years (n= 2404, unadjusted OR: 1.41, 95% CI 0.60 – 3.31, p=0.43; adjusted OR: 1.17, 95% CI 0.49 – 2.80, p=0.72). When examined as a binary variable there was some evidence of an association between paternal postnatal EPDS scores and offspring psychotic experiences in univariable models (n=2404, unadjusted OR: 2.41, 95% CI 1.06-5.49, p=0.035) but the strength of this evidence was attenuated after adjusting for confounders (adjusted OR: 2.25, 95% CI 0.98 – 5.17, p=0.055).

Schizophrenia polygenic risk score

Data on all exposures, outcomes, confounders and the schizophrenia PRS was available for 2347 of the study sample. There was evidence of an association between maternal antenatal EPDS scores and offspring psychotic experiences by the age of 18 years (unadjusted OR for a 5-point increase in EPDS score: 1.33, 95% CI 1.11 – 1.60, p=0.0021). There remained evidence for this association after adjusting for confounders, including the schizophrenia PRS (adjusted OR for a 5-point increase in EPDS score: 1.24, 95% CI 1.02 – 1.51, p=0.030). There was no evidence of an association between higher maternal postnatal EPDS scores and offspring psychotic experiences by the age of 18 years (unadjusted OR for a 5-point increase in EPDS scores: 1.16, 95% CI 0.95 – 1.40, p=0.14. OR for a 5-point increase in EPDS score after adjusting for confounders including schizophrenia PRS: 1.10, 95% CI 0.90 – 1.34, p=0.37).

Paternal family history of mental illness

In analyses adjusted for paternal family history of schizophrenia, paternal family history of depression and paternal depression in addition to the confounders the study sample size was reduced to 2392.

In this sample, we found evidence for an association between maternal antenatal depression and offspring psychotic experiences by the age of 18 years (unadjusted OR for a 5-point increase in EPDS score: 1.29, 95% CI 1.08 – 1.54, p=0.0007). The evidence for this association remained after adjustment for the confounders included in the main analyses (adjusted OR for a 5-point increase in EPDS score: 1.23, 95% CI 1.01 – 1.49, p=0.036) and after inclusion of additional confounders pertaining to paternal family history (adjusted OR for a 5-point increase in EPDS score: 1.22, 95% CI 1.00 – 1.48, p=0.049).

There was no evidence for an association between maternal postnatal depression and offspring psychotic experiences by the age of 18 years in this sample (unadjusted OR for a 5-point increase in EPDS score: 1.16, 95% CI 0.96-1.41, p=0.13. OR for a 5-point increase in EPDS score, adjusted for initial confounders: 1.13, 95% CI 0.93 – 1.39, p=0.22. OR for a 5-point increase in EPDS score, adjusted for paternal family history in addition to initial confounders: 1.12, 95% CI 0.91 – 1.37, p=0.29).

Family history of mania and mental health admission

In analyses adjusted for parental history of mania, parental family history of mania, parental history of mental health admission, and parental family history of mental health admission in addition to the confounders included in the main analyses, except for maternal cannabis use in the first trimester of pregnancy, the study sample size was 1967.

We found evidence for an association between maternal antenatal depression and offspring psychotic experiences by the age of 18 years (unadjusted OR for a 5-point increase in EPDS score: 1.50, 95% CI 1.22 – 1.85, p=0.0002). The evidence for this association remained after adjustment for the confounders included in the main analyses (adjusted OR for a 5-point increase in EPDS score: 1.40, 95% CI 1.12 – 1.75, $p<0.0030$) and after inclusion of the additional confounders relating to family history (adjusted OR for a 5-point increase in EPDS score: 1.41, 95% CI 1.13 – 1.77, p=0.0029).

We found evidence for an association between maternal postnatal depression and offspring psychotic experiences by the age of 18 years (unadjusted OR for a 5-point increase in EPDS score: 1.33, 95% CI 1.08 – 1.65, p=0.0076). The evidence for this association remained after adjustment for the confounders included in the main analyses (adjusted OR for a 5-point increase in EPDS score: 1.26, 95% CI 1.01 – 1.58, p=0.040) and after inclusion of the additional confounders relating to family history (adjusted OR for a 5-point increase in EPDS score: 1.30, 95% CI 1.04 – 1.63, p=0.023).

Supplementary References

1. Zammit S, Kounali D, Cannon M, David AS, Gunnell D, Heron J, et al. Psychotic experiences and psychotic disorders at age 18 in relation to psychotic experiences at age 12 in a longitudinal population-based cohort study. Am J Psychiatry. 2013/05/04. 2013;170(7):742–50.

2. Jones HJ, Stergiakouli E, Tansey KE, Hubbard L, Heron J, Cannon M, et al. Phenotypic Manifestation of Genetic Risk for Schizophrenia During Adolescence in the General Population. JAMA Psychiatry [Internet]. 2016;73(3):221. Available from: http://archpsyc.jamanetwork.com/article.aspx?doi=10.1001/jamapsychiatry.2015.3058

3. Purcell S, Neale B, Todd-Brown K, Thomas L, Ferreira MAR, Bender D, et al. PLINK: A tool set for whole-genome association and population-based linkage analyses. Am J Hum Genet. 2007;81(3):559–75.
